# ﻿*Middletonia tiepiana* (Gesneriaceae), a new species from southern Vietnam

**DOI:** 10.3897/phytokeys.267.169576

**Published:** 2025-12-12

**Authors:** Tran Quoc Trung Nguyen, Hieu Cuong Nguyen, Xuan Bach Nguyen-Le, Cong Luan Tran, Thanh Trung Nguyen, Hong Truong Luu

**Affiliations:** 1 Institute of Advanced Technology, Vietnam Academy of Science and Technology, Ho Chi Minh City, Vietnam Institute of Advanced Technology, Vietnam Academy of Science and Technology Ho Chi Minh City Vietnam; 2 Tay Do University, Can Tho City, Vietnam Tay Do University Can Tho City Vietnam; 3 Nui Chua National Park, Khanh Hoa Province, Vietnam Nui Chua National Park Khanh Hoa Province Vietnam; 4 Graduate University of Science and Technology, Vietnam Academy of Science and Technology, Hanoi, Vietnam Graduate University of Science and Technology Hanoi Vietnam

**Keywords:** Blue-violet flowers, endemic, Nui Chua National Park, semi-arid, UNESCO Biosphere Reserve

## Abstract

A new species of *Middletonia* (Gesneriaceae), *M. tiepiana*, is described from Nui Chua National Park in southern Vietnam. This is the third species of the genus recorded in Vietnam, and notably, the second Vietnamese species with a blue corolla – a trait previously observed in *M. evrardii* (Vietnam), *M. changjiangensis*, and *M. hainanensis* (both from China). *Middletonia tiepiana* differs from the Vietnamese species by its leaves lacking interpetiolar ridges, shorter peduncles, linear and minute bracts, free glandular anthers dehiscing by apical pores, and from the Chinese congeners by its growth form, distinct leaf morphology, length of peduncles, deep blue-violet corolla with a white base, and absence of staminodes. It grows in sandstone-based soils within coastal semi-arid forest, a habitat not previously associated with the genus. A detailed morphological description, illustration, notes on distribution, ecology, and phenology, and a provisional conservation assessment are provided. A key to all known *Middletonia* species is also included.

## ﻿Introduction

The genus *Middletonia* C.Puglisi belongs to the subtribe Loxocarpinae, tribe Trichosporeae, within the family Gesneriaceae (Weber et al. 2013). It was established by Puglisi et al. (2016) following a molecular phylogenetic study that revealed several species previously placed in *Paraboea* did not form a monophyletic group with the rest of the genus. This led to the segregation of *Middletonia*, now recognized as a distinct lineage supported by both molecular and morphological evidence.

Morphologically, *Middletonia* is distinguished from *Paraboea* by several key traits: erect anthers (vs. right-angled in *Paraboea*), minute glandular indumentum on the anthers and ovary (vs. glabrous or sparsely pubescent), conspicuous reticulate tertiary venation near leaf margins (vs. less distinct venation), smaller corolla, and shorter capsules (Puglisi et al. 2016; Puglisi and Middleton 2017; Phonepaseuth et al. 2021). Despite sharing several traits with *Paraboea* – such as matted indumentum on the abaxial leaf surface, flat-faced corolla (in some species), capsular fruits, persistent calyx lobes, cymose inflorescences, and lithophytic growth – these distinctions support the recognition of *Middletonia* as a separate genus within Loxocarpinae.

Species of *Middletonia* are perennial, lithophytic herbs typically found in shaded, moist limestone or granite habitats in India, Bangladesh, Bhutan, Myanmar, China, Thailand, Laos, Cambodia, Vietnam, and Malaysia (Puglisi and Middleton 2017). A revision of the genus in Thailand by Puglisi and Middleton (2017) clarified species boundaries and described *M. glebosa* as new, while resurrecting *M. reticulata* from synonymy. Consequently, five species were formally recognized within *Middletonia*: *M. evrardii* (Pellegr.) C.Puglisi, *M. glebosa* C.Puglisi, *M. multiflora* (R.Br.) C.Puglisi, *M. regularis* (Ridl.) C.Puglisi, and *M. reticulata* (Barnett) C.Puglisi. Most recently, based on molecular and morphological characteristics, Li et al. (2025) proposed the new combinations *M. changjiangensis* (F.W.Xing & Z.X.Li) X.X.Bai and *M. hainanensis* (Chun) X.X.Bai, bringing the total number of recognized species to seven. Vietnam is home to two species of *Middletonia*: *M. evrardii* and *M. multiflora* (Pellegrin 1930; Pham-Hoang 2000; Puglisi and Middleton 2017; Vu 2017). As botanical surveys in Vietnam remain incomplete, particularly in the central and northern limestone ranges, it is likely that additional species of *Middletonia* await discovery.

During recent botanical surveys in Nui Chua National Park, an undescribed species of *Middletonia* was discovered growing in crevices of sandstone-mixed soils within a coastal semi-arid forest. This habitat is notably distinct from the moist limestone or granite substrates typically associated with the genus, suggesting a broader ecological amplitude than previously recognized. Notably, this species exhibits blue-violet flowers – a trait previously observed only in *M. evrardii* from Vietnam and *M. changjiangensis* and *M. hainanensis* from China. After detailed morphological and comparative analysis, we conclude that the Vietnamese population represents a new species, which we describe below.

## ﻿Materials and methods

The studied material was collected from Nui Chua National Park, Khanh Hoa Province, southern Vietnam. Specimens were sampled and processed using methods described by the Royal Botanic Gardens, Kew (Bridson and Forman 1999). Herbarium acronyms follow Thiers (updated continuously). Detailed photographs and the description of taxonomically important characters of the new species were based on fresh material. Digital specimen images of compared species were examined from JSTOR Global Plants (https://plants.jstor.org/) and P (https://www.science.mnhn.fr/). Taxonomic identification was based on vegetative and reproductive morphological characters following the aforementioned literature.

## ﻿Taxonomic treatment

### ﻿ Middletonia tiepiana

Taxon classificationPlantaeLamialesGesneriaceae

Luu, X.B.Nguyen-Le & T.Q.T.Nguyen sp. nov.

61822382-3D4D-58B1-84F8-2C599E18B58B

urn:lsid:ipni.org:names:77373283-1

Fig. 1

#### Type.

Vietnam. Khanh Hoa Province, Nui Chua National Park, coordinates 11°42'30"N, 109°11'10"E, 100 m in elevation, 24 December 2024, *Luu Hong Truong, Nguyen Hieu Cuong Cu-2024-073* (holotype SGN! [SGN007460]; isotypes PHH!, BKF!, SGN! [SGN007459, SGN007462]).

#### Diagnosis.

*Middletonia tiepiana* is morphologically most similar to *M. evrardii* in its short caulescent habit, leaf shape, violet-blue corolla, floral structure, and twisted capsules, but differs by its smaller leaves (3.5–7 × 2.5–4 cm vs. 5–10 × 4–7 cm in *M. evrardii*), petiole densely brown tomentose and not interpetiolar ridged (vs. glabrescent, interpetiolar ridged), inflorescences with shorter peduncles (8–15 cm vs. 15–30 cm), linear bracts ca. 0.4 mm long (vs. lanceolate bracts 3–4 mm long), much shorter calyx lobes (1–1.4 mm vs. 3–4 mm), a corolla with a shorter tube (ca. 1 mm vs. 2 mm) and smaller lobes (4.6–6 × 3.7–4 mm vs. 6–7 × 5 mm), free and glandular anthers that are dehiscent by apical pores (vs. apically convergent, glabrous, dehiscent by longitudinal slits), smaller fruits (7.5–13 mm vs. 15–20 mm) and occurrence on semi-arid sandstone substrates (vs. moist granite rock).

#### Description.

Rhizomatous, perennial, shortly caulescent herb. Stem 3–7.5 cm tall. Leaves 7–10, tightly congested at the apex of stem, sub-opposite; petioles 1–4 cm long, densely brown tomentose; blades 3.5–7 × 2.5–4 cm, ovate or elliptic; apex obtuse to rounded; base obtuse, often oblique; margin irregularly crenate to crenulate; adaxially dark green and hispid; abaxially brown woolly with matted, interwoven long hairs, darker brown along the veins; venation impressed adaxially and prominent abaxially; secondary veins 5–7-paired, brochidodromous; tertiary venation clearly visible and reticulate. Inflorescence 2–3, axillary, compound cyme with numerous flowers, reddish brown tomentose, longer than the subtending leaf; peduncles 8–10 cm long, 1.5 mm in diameter; bracts linear, ca. 0.4 mm long, abaxially glandular; pedicels 1–13 mm long, tomentose. Calyx green, 5-lobed, tomentose and glandular outside; tube ca. 0.2 mm long; lobes narrowly triangular, 1–1.4 mm long, apex acute. Corolla almost flat-faced, ca. 1.35 × 1.3 cm, slightly bilabiate, sparsely glandular outside; tube short, ca. 1 mm long, deep blue-violet with a white base; lips deep blue-violet; upper lip ca. 5.1 × 7.2 mm, 2-lobed, lobes oblong to elliptic, ca. 4.7 × 3.7 mm; lower lip ca. 8 × 11 mm, 3-lobed, lobes oblong to elliptic, middle lobe ca. 4.6 × 3.8 mm, lateral lobes ca. 6 × 3.8–4 mm. Stamens 2, inserted at base of lower lip; filaments white, ca. 3 × 0.5 mm, straight, slightly curved at apex; anthers ovate, basifixed, yellow, ca. 2.2 × 1.8 mm, glandular, free, dehiscing by apical pores; pollen yellow; staminodes absent. Ovary pale green, ca. 2.2 mm long, 0.9 mm in diameter, densely covered with minuscule glands; ovules numerous, white; style 4.5–5.4 mm long, 0.3 mm in diameter, white, glabrous, straight; stigma capitate, white turning brown, slightly bilabiate, densely papillose. Capsule green, 7.5–13 mm long, 1.6–1.8 mm in diameter, densely glandular, 4-ridged, twisted, with persistent style. Seeds ovoid, 0.3–0.4 mm long, 0.15–0.18 mm in diameter.

**Figure 1.**
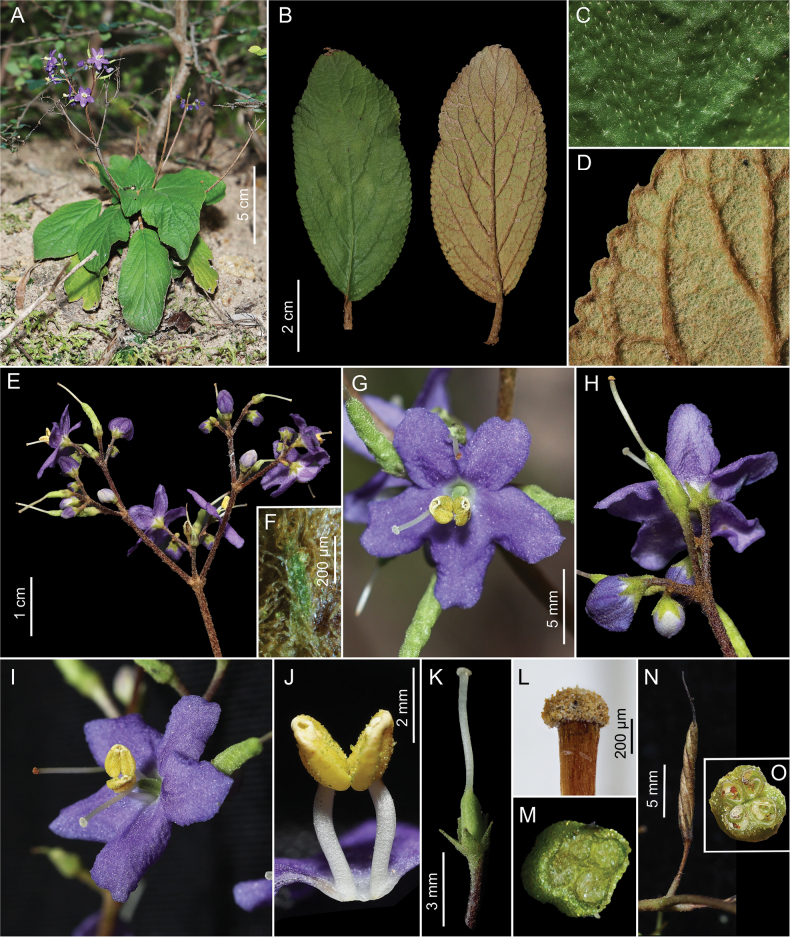
*Middletonia tiepiana*. **A.** Plant in situ; **B.** Leaves, adaxial and abaxial surfaces; **C.** Leaf, close-up of adaxial surface; **D.** Leaf, close-up of abaxial surface; **E.** Inflorescences; **F.** Bract; **G.** Flower, front view; **H.** Flower, dorsal view; **I.** Flower, side view; **J.** Stamens; **K.** Calyx & pistil; **L.** Close-up of stigma (dried); **M.** Cross-section of ovary; **N.** Fruit (immature); **O.** Cross-section of immature fruit.

#### Etymology.

The species is named in honor of Mr. Tran Van Tiep, Director of Nui Chua National Park, in recognition of his outstanding contributions to the conservation and stewardship of the park’s unique biodiversity.

#### Additional specimens examined.

***Middletonia evrardii*.** Vietnam, Lam Dong, Pongour pres Dijring [Di Linh], 24 August 1924, *Evrard 1177* (lectotype P, barcode [P00622885 photo seen]; isolectotypes P [P00556499 photo seen], [P00606306 photo seen, designated here). Vietnam. Lam Dong Province, Duc Trong ward, Pongour Water Fall, coordinates 12°09'38"N, 108°26'36"E, 821 m in elevation, 24 October, 2025; *Nguyen Tran Quoc Trung NTQT-1010* (SGN [SGN034025!, SGN034026!, SGN034027!]). ***Middletonia tiepiana*.** Vietnam. Khanh Hoa Province, Nui Chua National Park, coordinates 11°42'20"N, 109°11'31"E, 90 m in elevation, 01 October 2025, *Luu Hong Truong & Nguyen Thanh Trung Luu 3001* (paratypes SGN! [SGN034003, SGN034004, SGN034005], VNMN!, HN!).

#### Vernacular name.

*Song bế Tiếp* (Vietnamese).

#### Phenology.

Flowering and fruiting observed from October to January.

#### Distribution and ecology.

*Middletonia tiepiana* is currently known only from Nui Chua National Park, Khanh Hoa Province, southern Vietnam. The species was discovered growing in crevices of sandstone-mixed soils within coastal semi-arid forest – a habitat characterized by prolonged seasonal drought, intense solar radiation, and sparse canopy cover. The region receives an average annual rainfall of less than 800 mm, concentrated primarily between late September and mid-December. This area lies within the “Southern Vietnam Lowland Dry Forests” ecoregion, considered the most arid and ecologically distinctive ecosystem in Vietnam, and across Southeast Asia (Thai 1999; Wikramanayake et al. 2002). The vegetation at the collection site is dominated by drought-adapted species such as *Strychnos nux-vomica*, *Buchanania reticulata*, *Diospyros mun*, *Milletia* sp., *Morinda cochinchinensis*, *Terminalia chebula*, and *Spondias pinnata*. This sandstone-based coastal dry forest represents a unique habitat within the known distribution of *Middletonia*, and may serve as a relictual refugium for Gesneriaceae lineages adapted to xeric conditions. The distribution of *M. tiepiana* appears to be restricted to this specialized ecosystem.

#### Preliminary IUCN conservation status.

Data Deficient (IUCN Standards and Petitions Subcommittee 2024). The species appears to be rare, with only two small populations observed at the type locality. Its actual distribution and population size remain unknown, though it may occur in similar habitats south of Nui Chua National Park. Further field surveys are required to determine its full extent and conservation status. Although the known population lies within a protected area, the species’ narrow ecological niche and limited population size render it highly vulnerable to stochastic events and habitat shifts. Conservation measures should prioritize in situ protection, including regular monitoring of known individuals and habitat management to mitigate anthropogenic pressures. Ex situ conservation, such as seed banking and cultivation trials, may be considered to safeguard against potential extinction

#### Taxonomic notes.

**Figure 2.**
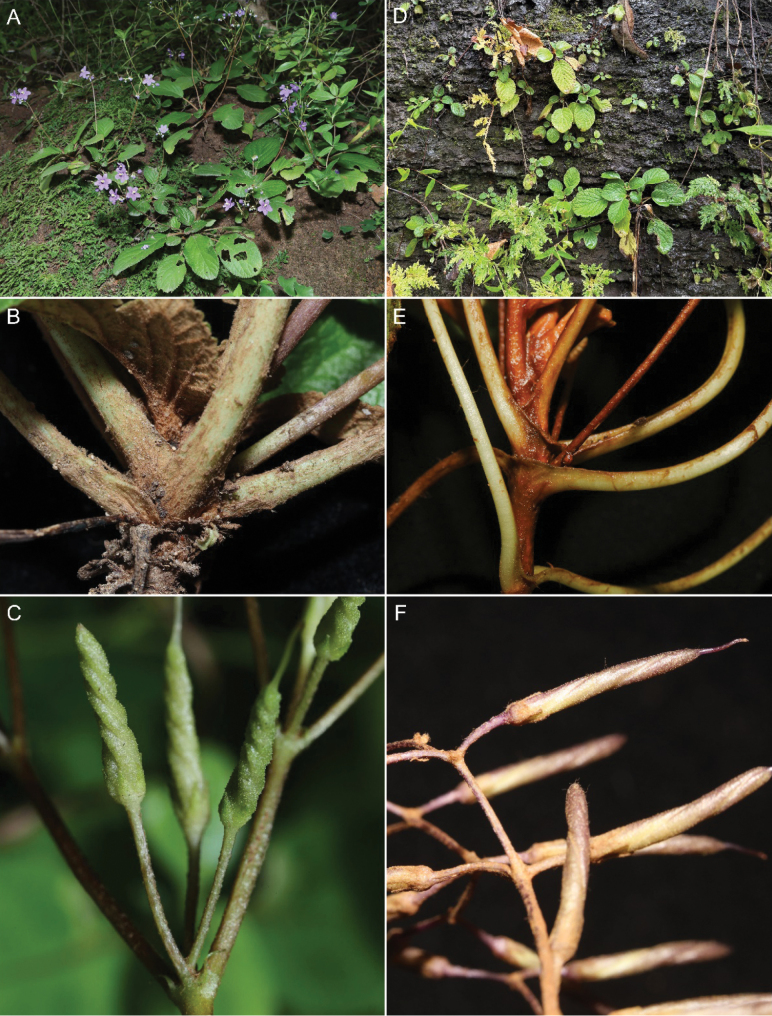
Comparison of *Middletonia tiepiana* (**A–C**) and *M. evrardii* (**D–F**). **A.** Habitat. **B.** Interpetiolar ridge. **C.** Fruits.

*Middletonia evrardii* was first described as *Boea evrardii*Pellegrin (1930), based on *Evrard 1177* (P) collected at Pongour Waterfall near Di Linh, Lam Dong Province, in the Central Highlands of Vietnam. Pellegrin characterized the species as a short-stemmed herb with opposite oblong leaves, violet corolla, and strongly twisted capsules. Examination of the scanned type specimens (JSTOR Global Plants 2025) and our field observations confirm that its leaf blades are variable (elliptic, elliptic to obovate) rather than oblong, and petioles are glabrescent with interpetiolar ridges (Fig. 2). The type locality is situated on moist granite outcrops, not limestone as originally noted by Evrard.

Xu et al. (2008) later broadened the circumscription of *Paraboea evrardii* (now *M. evrardii*) by including several historical collections from central and south-central Vietnam, though without detailed morphological comparison to the type. Among these, *Hayata 738* (P [P00634329], Da Ban, Quang Ngai Province, 18 June 1921) was reassigned from Boea multiflora R.Br. var. villosa Pellegrin to *P. evrardii*. Our examination of *Hayata 738* via a scanned image (JSTOR Global Plants 2025) reveals notable differences from the type, supporting Xu et al.’s suggestion that “the status of this taxon may need to be reviewed in the future with more collections.” *Hayata 738* bears leaves that are basally attenuate and proportionally narrower than those of the types (*Evrard 1177*, P [P00622885, P00556499, P00606306]), which are basally oblique and obtuse, broader and often nearly as wide as long. Both surfaces are densely pubescent, with the abaxial side covered by a very thick, dark-brown matted indumentum – among the densest observed in the genus. Petioles are shorter (2–4 cm vs. 3–8 cm). Inflorescences have shorter peduncles (10–15 cm vs. 25–30 cm) but longer axes (1.5–2.5 cm) and pedicels (ca. 1 cm), with smaller calyx lobes. The extremely thick indumentum may reflect adaptation to drier, more exposed microhabitats. These observations align with Pellegrin’s (1930) notes that B. multiflora var. villosa is characterized with lanceolate leaves, less obtuse, densely woolly even between the veins. Although *Hayata 738* shares certain vegetative traits with *M. tiepiana*, it differs markedly in leaf shape, indumentum density, floral proportions, and capsule size, and was collected from a more humid montane granite habitat. These differences indicate that *Hayata 738* does not represent *M. tiepiana* nor *M. evrardii* s.str., but rather a morphological and ecological variant within the *M. evrardii* complex.

Three additional specimens cited by Xu et al. (2008) – *D’Alleizette s.n.* (Phan Rang, Ninh Thuan Province, June 1909; L), *Poilane 20888* (Phan Rang, 20 August 1922; P), and *Poilane 30612* (Song Cay Valley, 28 September 1940; P) – could not be examined directly, and no digital images are currently available. Their identity remains uncertain; they may correspond to *M. evrardii* s.str., *M. tiepiana*, or another taxon distributed along the south-central Vietnam aridity gradient.

In the Thai revision of *Middletonia*, Puglisi and Middleton (2017) treated *M. evrardii* as a white-flowered species from Thailand and Laos. This interpretation differs from both Pellegrin’s violet-flowered type and Xu et al.’s broader Vietnamese concept. While Puglisi & Middleton acknowledged the type specimen (*Evrard 1177*), they did not discuss the other Vietnamese collections reassigned by Xu et al. (2008). It was additionally noted by Puglisi and Middleton (2017) that Thai and Vietnamese plants exhibited differences in leaf shape and further material is required to resolve species limits. Until such data are available, the circumscription of *M. evrardii* across Indochina remains uncertain. Although flower color in Gesneriaceae can range from white to violet or blue within a single species and may be thus of limited diagnostic value, we follow Pellegrin’s original concept based on *Evrard 1177* and treat the Thai white-flowered taxon as distinct, pending a comprehensive review.

As such, *M. evrardii* s.str. is a violet-blue-flowered species confined to humid granite habitats (Fig. 2) of the Central Highlands of Vietnam at around 800 m elevation. This interpretation is consistent with Vu (2017), who did not include *Hayata 738*, *D’Alleizette s.n.*, *Poilane 20888* and *Poilane 30612* under *P. evrardii*.

*M. tiepiana* is morphologically closest to *M. evrardii* s.str. in habit, leaf shape, corolla color, floral structure, and twisted capsules. However, it occupies semi-arid sandstone substrates at approximately 100 m elevation. In the absence of reproductive material, *M. evrardii* can be distinguished from *M. tiepiana* by its opposite leaves with interpetiolar ridges and glabrescent petioles. The fruits of *M. evrardii* are brownish-purplish while those of *M. tiepiana* are green. These characters are illustrated in Fig. 2. Additional diagnostic characters are summarized in the species diagnosis.

*Middletonia multiflora* resembles *M. tiepiana* in having a short caulescent or rhizomatous habit, petiolate leaves with dense abaxial indumentum, and twisted capsules. However, it differs markedly in several traits: its leaf blades are substantially larger (up to 22 cm vs. up to 7 cm), typically arranged in a basal rosette or along an elongate rhizome, and have cuneate leaf bases with glabrescent or rarely pubescent petioles, contrasting with the seemingly opposite and tightly congested leaves with oblique bases and densely brown tomentose petioles in *M. tiepiana*. The corolla of *M. multiflora* is white, while *M. tiepiana* has a deep blue-violet corolla with a white base. Additionally, *M. multiflora* inhabits moist limestone up to 1300 m elevation, whereas *M. tiepiana* grows on semi-arid sandstone substrates at around 100 m.

*Middletonia tiepiana* is also distinguished from the two blue-violet Chinese species *M. changjiangensis* and *M. hainanensis*. It differs from *M. changjiangensis* in several key characters: rhizomatous habit with short stems (3–7.5 cm vs. 9–40 cm); tightly congested leaf arrangement (vs. leaves spread along the stem); compound cymose inflorescence (vs. simple cymose); longer peduncles (8–10 cm vs. 4–5 cm); deep blue-violet corolla with a white base (vs. pale blue); absence of staminodes (vs. two present); densely glandular ovary (vs. glabrous or farinose glandular); and densely glandular capsule (vs. glabrous). *Middletonia hainanensis* differs from the new species in a number of key characters: its leaves are sessile oblanceolate to obovate with attenuate bases (vs. ovate to elliptic, petiolate leaves with unequal bases in *M. tiepiana*), peduncles are longer (12–30 cm vs. 8–10 cm), and its reproductive structures are less densely glandular. It also occurs on limestone at ~800 m elevation, whereas *M. tiepiana* grows on semi-arid sandstone substrates at ~100 m elevation.

Ecologically, *M. tiepiana* is notable for its occurrence in sandstone-based soil crevices within coastal semi-arid forest – a habitat not previously associated with *Middletonia*, which is typically found on moist limestone or granite substrates. This suggests a broader ecological amplitude for the genus than previously recognized and highlights the importance of underexplored habitats in revealing hidden botanical diversity. The discovery of *M. tiepiana* in Nui Chua National Park further features the conservation significance of this region, recently designated as Vietnam’s 11^th^ UNESCO Biosphere Reserve, home to a distinctive flora uniquely adapted to extreme environmental conditions (UNESCO 2022). Several new endemic species have recently been described from this ecosystem, including *Aristolochia thotteaeformis* T.V.Do & Luu (Luu et al. 2022), *Balanites vietnamica* Luu, Th.Trung Nguyen & T.Q.T.Nguyen (Nguyen et al. 2023), *Memecylon longipedunculatum* Tagane, V.S.Dang & Nuraliev (Tagane et al. 2025b) and *Wrightia nuichuaensis* Tagane & V.S.Dang (Tagane et al. 2025a).

A detailed morphological comparison among *Middletonia tiepiana* and its closest congeners is presented in Table 1.

**Table 1.** Comparison of *Middletonia tiepiana* with its morphologically close congeners (based on Pellegrin 1930; Wang et al. 1998; Xu et al. 2008; Li et al. 2025; and our field observations).

Character	*M. tiepiana*	*M. changjiangensis*	*M. evrardii*	*M. hainanensis*	*M. multiflora*
Stem	3–7.5 cm high	9–40 cm high	5–6 cm high	to 5 cm high	to 20 cm high
Leaves	sub-opposite, tightly congested, petiolate	opposite, arranged along stem, petiolate	opposite, arranged along stem, petiolate	tightly congested, sessile	tightly congested or arranged along stem, petiolate
Leaf blade	ovate to elliptic, 3.5–7 × 2.5–4 cm	elliptic to oblong, 2–7 × 1–3 cm	ovate, elliptic or oblanceolate, 5–10 × 4–7 cm	oblanceolate to obovate, 5–18 × 1.2–6 cm	elliptic to rarely ovate, 4–22 × 2.5–9 cm
Leaf base	obtuse, often oblique	cuneate	obtuse	gradually attenuate	cuneate, rarely slightly cordate
Petiole	densely brown tomentose	unknown	glabrescent	Unknown	glabrescent or rarely pubescent
Interpetiolar ridge	Absent	absent	present	Absent	absent
Inflorescence	compound cyme	simple cyme	compound cyme	compound cyme	compound cyme
Peduncle	8–10 cm long	4–5 cm long	15–30 cm long	12–30 cm long	3.5–22 cm long
Corolla color	deep blue-violet with white base	pale purple	blue-violet	bluish to deep purple	white
Anthers	glandular, free	glabrous, coherent	glabrous, apically convergent	glabrous, coherent	farinose, free
Staminodes	Absent	2	absent	absent or 1	absent
Ovary	densely glandular	glabrous or farinose glandular	glandular	Puberulent	farinose
Capsule	7.5–13 mm, glandular	8–15 mm, glabrous	15–20 mm, glandular	3–4.5 cm, glabrous	6–12 mm, farinose
Habitat	semi-arid sandstone-mixed soils, at ~100 m in elevation	moist limestone, at ~600 m in elevation	moist granite, at ~800 m in elevation	shady moist rocks, at ~800 m in elevation	moist limestone, 100–1300 m in elevation
Distribution	Southern Vietnam	Hainan (China)	Central Highlands, Vietnam	Hainan (China)	Eastern Himalaya to China and Indochina

### ﻿Key to the 8 presently known species of *Middletonia*

Based on Pellegrin 1930; Wang et al. 1998; Pham-Hoang 2000; Puglisi and Middleton 2017; Vu 2017; Li et al. 2025.

**Table:** 

1	Inflorescence longer than the subtending leaf; fruit strongly twisted	**2**
–	Inflorescence shorter than or as long as the subtending leaf; fruit straight or slightly twisted	**7**
2	Leaf sessile	***M. hainanensis***
–	Leaf petiolate	**3**
3	Leaves densely pubescent above	**4**
–	Leaves glabrous or glabrescent above	**6**
4	Stems 9–40 cm long; leaf base cuneate	***M. changjiangensis***
–	Stems 3–7.5 cm long; leaf base unequally obtuse	**5**
5	Leaves interpetiolar ridged; petioles densely brown tomentose	***M. evrardii***
–	Leaves not interpetiolar ridged; petioles glabrescent	***M. tiepiana***
6	Leaf with oblique base and loose indumentum abaxially	***M. reticulata***
–	Leaf with cuneate base and dense indumentum abaxially	***M. multiflora***
7	Fruit slightly twisted, valves smooth; habit distinctly caulescent	***M. regularis***
–	Fruit straight, valves lumpy; habit shortly caulescent	***M. glebosa***

## Supplementary Material

XML Treatment for Middletonia tiepiana

## ﻿Additional information

### Use of AI

No use of AI was reported.

### Author ORCIDs

Tran Quoc Trung Nguyen https://orcid.org/0000-0003-4366-2381

Hieu Cuong Nguyen https://orcid.org/0000-0001-8707-2944

Xuan Bach Nguyen-Le https://orcid.org/0000-0001-8391-338X

Cong Luan Tran https://orcid.org/0009-0002-2202-0654

Thanh Trung Nguyen https://orcid.org/0000-0001-5546-6914

Hong Truong Luu https://orcid.org/0000-0002-7036-7081

### Data availability

All of the data that support the findings of this study are available in the main text.
